# A new species of the genus *Odorrana* Fei, Ye & Huang, 1990 (Anura, Ranidae) from southeastern Yunnan, China

**DOI:** 10.3897/zookeys.1275.186067

**Published:** 2026-03-26

**Authors:** Shuo Liu, Chao Bu, Yanfei Feng, Mian Hou, Dingqi Rao, Song Li

**Affiliations:** 1 Kunming Natural History Museum of Zoology, Kunming Institute of Zoology, Chinese Academy of Sciences, Kunming, Yunnan 650223, China Sichuan Normal University Chengdu China; 2 Yunnan Key Laboratory of Biodiversity Information, Kunming Institute of Zoology, Chinese Academy of Sciences, Kunming, Yunnan 650201, China Kunming Natural History Museum of Zoology, Kunming Institute of Zoology, Chinese Academy of Sciences Kunming China; 3 Yunnan Wenshan National Nature Reserve Management and Protection Bureau, Wenshan, Yunnan 663000, China Yunnan Key Laboratory of Biodiversity Information, Kunming Institute of Zoology, Chinese Academy of Sciences Kunming China; 4 Wenshan City Management and Protection Sub-Bureau of Yunnan Wenshan National Nature Reserve, Wenshan, Yunnan 663000, China Yunnan Wenshan National Nature Reserve Management and Protection Bureau Wenshan China; 5 College of Continuing (Online) Education, Sichuan Normal University, Chengdu, Sichuan 610066, China Wenshan City Management and Protection Sub-Bureau of Yunnan Wenshan National Nature Reserve Wenshan China; 6 Kunming Institute of Zoology, Chinese Academy of Sciences, Kunming, Yunnan 650201, China Kunming Institute of Zoology, Chinese Academy of Sciences Kunming China

**Keywords:** 16S rRNA, morphology, phylogeny, taxonomy, Wenshan Prefecture

## Abstract

A new species of the genus *Odorrana* is described based on specimens collected from Wenshan Prefecture, Yunnan Province, China. The new species can be distinguished from other species of the genus by a combination of the following characteristics: snout–vent length 43.5–46.9 mm in males, females approximately two times size of males, head length greater than head width, nostril closer to tip of snout than to eye, tibiotarsal articulation reaching tip of snout when hindlimb stretched forward, relative lengths of fingers III > IV > I > II, dorsolateral fold absent, anterior dorsum green with evenly distributed small irregular shaped black blotches and posterior dorsum greyish brown with evenly distributed, large, irregular-shaped, black blotches, external vocal sacs present in adult males. In addition, the new species differs from its congeners by a genetic distance of 3.6%–14.2% in the mitochondrial 16S rRNA gene.

## Introduction

The genus *Odorrana* Fei, Ye & Huang, 1990, commonly known as odorous frogs, is one of the most speciose groups of ranids whose range covers Japan, southern China, northeastern India and almost the whole Southeast Asia (Kilunda et al. 2025; Li et al. 2025; Frost 2026). This genus currently contains 70 species, and nearly 70% of these occur in southern China (AmphibiaChina 2026; Frost 2026). New species of *Odorrana* are continuously being described, which indicates that the diversity of this genus is still underestimated.

The new species of the genus *Odorrana* described in recent years are all from China and mainly concentrated in the *O. andersonii* (Boulenger, 1882) group (*O. dulongensis* Liu, Che & Yuan, 2021 and *O. sudianensis* Kilunda, Yu, Wu & Che, 2025), the *O. schmackeri* (Boettger, 1892) group (*O. fengkaiensis* Wang, Lau, Yang, Chen, Liu, Pang & Liu, 2015, *O. kweichowensis* Li, Xu, Lv, Jiang, Wei & Wang, 2018, *O. ichangensis* Chen, 2020, and *O. sangzhiensis* Zhang, Li, Hu & Yang, 2021), the *O. versabilis* (Liu & Hu, 1962) group (*O. confusa* Song, Zhang, Qi, Lyu, Zeng & Wang, 2023, and *O. damingshanensis* Chen, Mo, Lin & Qin, 2024), and the *O. lipuensis* Mo, Chen, Wu, Zhang & Zhou, 2015 group (*O. lipuensis*, *O. liboensis* Luo, Wang, Xiao, Wang & Zhou, 2021, *O. concelata* Wang, Zeng & Lin, 2022, *O. calciphila* Song, Qi, Wang, Liu & Wang, 2025, and *O. feii* Li, Mu, Jing, Liu, Cheng & Wang, 2025) (Frost 2026). Until now, 47 species of *Odorrana* have been recorded in China, and 10 of these are from the region near the border between China and Vietnam (AmphibiaChina 2026).

The China–Vietnam border region is an extremely biodiverse area, which has an intact forest ecosystem and diverse habitats, resulting in its high species richness (Myers et al. 2000; Mittermeier et al. 2011; Das and van Dijk 2013; De Bruyn et al. 2014; Poyarkov et al. 2021). In recent years, many new species of amphibians have been found from this region, including *Zhangixalus franki* Ninh, Nguyen, Orlov, Nguyen & Ziegler, 2020; *Micryletta hekouensis* Liu, Hou, Mo & Rao, 2021; *Amolops shihaitaoi* Wang, Li, Du, Hou & Yu, 2022; *Paramesotriton malipoensis* Rao, Liu, Zhu & Ma, 2022; *Rhacophorus napoensis* Li, Liu, Yu & Sun, 2022; *Rh. trangdinhensis* Kropachev, Evsyunin, Orlov & Nguyen, 2022; *Theloderma hekouense* Du, Wang, Liu & Yu, 2022; *T. khoii* Ninh, Nguyen, Nguyen, Hoang, Siliyavong, Nguyen, Le, Le & Ziegler, 2022; *Raorchestes malipoensis* Huang, Liu, Du, Bernstein, Liu, Yang, Yu & Wu, 2023; *Ra. hekouensis* Du, Xu, Liu & Yu, 2024; *Ichthyophis yangi* Rao, Zhou, Mo, Yu, Li & Liu, 2024; *Tylototriton koliaensis* Poyarkov, Nguyen, Le, Le, Arkhipov, Gorin, Hernandez & Dufresnes, 2024; *Quasipaa phamanhi* Liu, Zhu, Pham, Hoang, Nguyen, Vu, Hou, Mo & Rao, 2025; *Rh. hujianshengi* Liu, Hou, Rao & Li, 2025; *Z. daweishanensis* Pan, Liu, Chen & Yu, 2025; and so on (Frost 2026).

During our recent field surveys in southern Yunnan Province, China, some specimens of the genus *Odorrana* were collected in Funing County, Wenshan Prefecture, Yunnan Province, China, from the region near the border between China and Vietnam. Subsequent morphological and molecular analyses identified these specimens to be an unnamed species. Herein, we describe it as a new species of *Odorrana*.

## Materials and methods

Specimens were collected by hand and preserved in 75% ethanol. Photographs were taken in life after the frogs were collected. All specimens were deposited at Kunming Natural History Museum of Zoology, Kunming Institute of Zoology, Chinese Academy of Sciences (**KIZ**).

Total genomic DNA was extracted from liver tissues of the specimens. A fragment of the mitochondrial 16S rRNA gene was amplified via the polymerase chain reaction (PCR) using the primers L2188 (Matsui et al. 2006) and 16H1 (Hedges 1994). Molecular experiment protocols used in this study were the same as those in Liu et al. (2022). All new sequences have been deposited on GenBank, and other sequences were downloaded from GenBank (Table 1). Sequences of *Nidirana daunchina* (Chang, 1933), *Pelophylax nigromaculatus* (Hallowell, 1861), and *Amolops loloensis* (Liu, 1950) were used as outgroups according to Kilunda et al. (2025). The technical computation methods for sequence alignment, best substitution model selection, Bayesian-inference and maximum-likelihood phylogenetic analysis, and genetic divergences calculation were in the same manner as by Liu et al. (2022).

**Table 1.** Samples used in the phylogenetic analysis of this study.

Species	Locality	Voucher	Accession	Reference
*Odorrana yangi* sp. nov.	Funing, Yunnan, China	KIZ2025123	PX979645	This study
*Odorrana yangi* sp. nov.	Funing, Yunnan, China	KIZ2025124	PX979646	This study
*Odorrana yangi* sp. nov.	Funing, Yunnan, China	KIZ2025143	PX979647	This study
*Odorrana yangi* sp. nov.	Funing, Yunnan, China	KIZ2025144	PX979648	This study
*Odorrana yangi* sp. nov.	Funing, Yunnan, China	KIZ2025145	PX979649	This study
*Odorrana yangi* sp. nov.	Funing, Yunnan, China	KIZ2025146	PX979650	This study
*Odorrana yangi* sp. nov.	Funing, Yunnan, China	KIZ2025147	PX979651	This study
*Odorrana yangi* sp. nov.	Funing, Yunnan, China	KIZ2025148	PX979652	This study
*Odorrana amamiensis*	Kagoshima, Japan	KUHE 29529	AB200946	Matsui et al. (2005)
*Odorrana andersonii*	Longchuan, Yunnan, China	HNNU001YN	KF185057	Chen et al. (2013)
*Odorrana anlungensis*	Anlong, Guizhou, China	HNNU1008I109	KF185049	Chen et al. (2013)
*Odorrana aureola*	Phu Ruea, Loei, Thailand	FMNH 265925	DQ650568	Stuart et al. (2006)
*Odorrana bacboensis*	Khe Moi, Nghe An, Vietnam	ROM 13044	DQ650569	Stuart et al. (2006)
*Odorrana bacboensis*	Napo, Guangxi, China	SYS a001046	KT315385	Wang et al. (2015)
*Odorrana banaorum*	An Khe, Gia Lai, Vietnam	ROM 39913	DQ650586	Stuart et al. (2006)
*Odorrana calciphila*	Yangshan, Guangdong, China	SYS a008923	PV347065	Song et al. (2025)
*Odorrana chapaensis*	Van Ban, Lao Cai, Vietnam	AMNH A161439	DQ283372	Frost et al. (2006)
*Odorrana chloronota*	Medog, Xizang, China	SYS a008274	PV347071	Song et al. (2025)
*Odorrana concelata*	Qingyuan, Guangdong, China	GEP a050	OP137167	Lin et al. (2022)
*Odorrana confusa*	Shixing, Guangdong, China	SYS a008595	OR658982	Song et al. (2023)
*Odorrana damingshanensis*	Wuming, Guangxi, China	NNU00690	ON791420	Chen et al. (2024)
*Odorrana dulongensis*	Gongshan, Yunnan, China	KIZ035027	MW128102	Liu et al. (2021)
*Odorrana exiliversabilis*	Wuyishan, Fujian, China	HNNU0607032	KF185056	Chen et al. (2013)
*Odorrana feii*	Xuwen, Guizhou, Chian	MT XW20250504001	PV951782	Li et al. (2025)
*Odorrana fengkaiensis*	Fengkai, Guangdong, China	SYS a002262	KT315375	Wang et al. (2015)
*Odorrana fengkaiensis*	Fengkai, Guangdong, China	SYS a002273	KT315377	Wang et al. (2015)
*Odorrana geminata*	Cao Bo, Ha Giang, Vietnam	AMNH 163782	EU861546	Bain et al. (2009)
*Odorrana grahami*	Kunming, Yunnan, China	HNNU1008-016	KF185051	Chen et al. (2013)
*Odorrana graminea*	Wuzhishan, Hainan, China	HNNU0606123	KF185038	Chen et al. (2013)
*Odorrana hainanensis*	Wuzhishan, Hainan, China	HNNU0606105	KF185032	Chen et al. (2013)
*Odorrana hainanensis*	Lingshui, Hainan, China	SYS a002260	KT315383	Wang et al. (2015)
*Odorrana heatwolei*	Phongsaly, Phongsaly, Laos	FMNH 258134	OR237216	Liu et al. (2023)
*Odorrana hejiangensis*	Chishui, Guizhou, China	MT CS20200605008	OR879764	Li et al. (2024)
*Odorrana hejiangensis*	Hejiang, Sichuan, China	HNNU1007-202	KF185052	Chen et al. (2013)
*Odorrana hosii*	Kuala Lumpur, Malaysia	IABHU 21004	AB511284	Kurabayashi et al. (2010)
*Odorrana huanggangensis*	Wuyishan, Fujian, China	SYS a004148	OR658951	Song et al. (2023)
*Odorrana huanggangensis*	Wuyishan, Fujian, China	HNNU0607001	KF185059	Chen et al. (2013)
*Odorrana ichangensis*	Zhijin, Guizhou, China	MT ZJ20210814004	OR879767	Li et al. (2024)
*Odorrana ichangensis*	Yichang, Hubei, China	SYS a005475	OR879765	Li et al. (2024)
*Odorrana ishikawae*	Okinawa, Japan	KUHE 10069	AB200945	Matsui et al. (2005)
*Odorrana jingdongensis*	Jingdong, Yunnan, China	20070711017	KF185050	Chen et al. (2013)
*Odorrana junlianensis*	Junlian, Sichuan, China	HNNU002JL	KF185058	Chen et al. (2013)
*Odorrana kuangwuensis*	Nanjiang, Sichuan, China	HNNU0908I185	KF185034	Chen et al. (2013)
*Odorrana kweichowensis*	Jinsha, Guizhou, China	CIBjs20150803008	MH193552	Li et al. (2018)
*Odorrana kweichowensis*	Jinsha, Guizhou, China	CIBjs20171014001	MH193551	Li et al. (2018)
*Odorrana leishanensis*	Leishan, Guizhou, China	MT LS20230806010	OR879754	Li et al. (2024)
*Odorrana leishanensis*	Leishan, Guizhou, China	MT LS20230729013	OR879757	Li et al. (2024)
*Odorrana leporipes*	Shaoguan, Guangdong, China	HNNU1008-099	KF185036	Chen et al. (2013)
*Odorrana liboensis*	Libo, Guizhou, China	GZNU20180608007	MW481350	Luo et al. (2021)
*Odorrana lipuensis*	Lipu, Guangxi, China	NHMG1303018	MH665676	Mo et al. (2015)
*Odorrana livida*	Thagata Juwa, Myanmar	BMNH 1889.3.25.48	DQ650615	Stuart et al. (2006)
*Odorrana lungshengensis*	Longsheng, Guangxi, China	HNNU70028	KF185054	Chen et al. (2013)
*Odorrana macrotympana*	Yingjiang, Yunnan, China	KIZ 2009051020	OL831010	Liu et al. (2022)
*Odorrana margaretae*	Dujiangyan, Sichuan, China	SYS a003214	KT315391	Wang et al. (2015)
*Odorrana morafkai*	Tram Lap, Vietnam	ROM 7446	AF206484	Chen et al. (2005)
*Odorrana mutschmanni*	Cao Bang, Vietnam	IEBR 3725	KU356766	Pham et al. (2016b)
*Odorrana nanjiangensis*	Nanjiang, Sichuan China	HNNU1007-291	KF185042	Chen et al. (2013)
*Odorrana narina*	Okinawa, Japan	/	AB511287	Kurabayashi et al. (2010)
*Odorrana nasica*	Ha Tinh, Vietnam	AMNH A161169	DQ283345	Frost et al. (2006)
*Odorrana nasuta*	Wuzhishan, Hainan, China	HNNU051119	KF185053	Chen et al. (2013)
*Odorrana sangzhiensis*	Sangzhi, Hunan, China	CSUFT 4308220046	MW464864	Zhang et al. (2021)
*Odorrana sangzhiensis*	Sangzhi, Hunan, China	CSUFT 4308220051	MW464865	Zhang et al. (2021)
*Odorrana schmackeri*	Yichang, Hubei, China	HNNU0908II349	KF185047	Chen et al. (2013)
*Odorrana schmackeri*	Songtao, Guizhou, China	MT ST20210622002	OR879769	Li et al. (2024)
*Odorrana splendida*	Kagoshima, Japan	IABHU 5275	AB511282	Kurabayashi et al. (2010)
*Odorrana sudianensis*	Yingjiang, Yunnan, China	KIZ 058904	LC902022	Kilunda et al. (2025)
*Odorrana supranarina*	Okinawa, Japan	KUHE 12898	AB200950	Matsui et al. (2005)
*Odorrana swinhoana*	Nantou, Taiwan, China	HNNUTW9	KF185046	Chen et al. (2013)
*Odorrana tianmuii*	Lin’an, Zhejiang, China	HNNU707071	KF185040	Chen et al. (2013)
*Odorrana tianmuii*	Lin’an, Zhejiang, China	SYS a002680	OR879761	Li et al. (2024)
*Odorrana tiannanensis*	Hekou, Yunnan, China	KIZ20193272	OL831009	Liu et al. (2022)
*Odorrana tormota*	Huangshan, Anhui, China	AM04005	DQ835616	Su et al. (2007)
*Odorrana versabilis*	Leishan, Guizhou, China	HNNU003	KF185055	Chen et al. (2013)
*Odorrana wuchuanensis*	Wuchuan, Guizhou, China	HNNU019L	KF185043	Chen et al. (2013)
*Odorrana yentuensis*	Vietnam	IEBR A.2015.38	KX893891	Unpublished
*Odorrana yizhangensis*	Yizhang, Hunan, China	HNNU1008I075	KF185048	Chen et al. (2013)
*Amolops loloensis*	Shimian, Sichuan, China	SM-ZDTW-01	NC_029250	Xue et al. (2016)
*Nidirana daunchina*	Emeishan, Sichuan, China	HNNU20060103	KF185065	Chen et al. (2013)
*Pelophylax nigromaculata*	Hongya, Sichuan, China	SCUM045199CJ	KX269216	Yuan et al. (2016)

Measurements were taken with digital callipers to the nearest 0.1 mm. Morphological characters followed Li et al. (2024):

**ED** eye diameter, between two corners of eye;

**FL** foot length, from proximal edge of inner metatarsal tubercle to tip of fourth toe;

**HDL** head length, from tip of snout to articulation of jaw;

**HDW** head width, maximum width between two articulations of jaw;

**HLL** hindlimb length, from vent to tip of fourth toe;

**IND** internasal distance, between inner margins of each nostril;

**IOD** minimum distance between inner edges of each upper eyelid;

**LAL** lower arm and hand length, from elbow to tip of third finger;

**ML** manus length, from proximal edge of inner palmar tubercle to tip of third finger;

**NED** nostril to eye distance, between nasal and anterior corner of eye;

**NSD** nostril to snout distance, between nasal and tip of snout;

**SVL** snout-vent length, from tip of snout to posterior edge of vent;

**SL** snout length, from tip of snout to anterior corner of eye;

**TFL** foot and tarsus length, from tibiotarsal articulation to tip of fourth toe;

**THL** thigh length, from vent to knee;

**TL** tibia length, from knee to tibiotarsal articulation;

**TYD** maximum diameter of tympanum;

**UEW** maximum width of upper eyelid.

For morphological comparison, the morphological data of other species of the genus were obtained from the original or subsequent descriptions of the species (Boulenger 1900, 1903, 1917, 1920; Stejneger 1901; Liu 1950; Liu and Hu 1962; Hu et al. 1966, 1973; Pillai and Chanda 1977; Wu 1977; Yang and Li 1980; Wu et al. 1983; Deng and Yu 1992; Matsui 1994; Fei et al. 2001a, 2001b, 2007a, 2007b, 2009; Bain et al. 2003, 2009; Stuart and Bain 2005; Stuart and Chan-ard 2005; Stuart et al. 2005, 2006; Bain and Stuart 2006; Matsui and Jaafar 2006; Orlov et al. 2006; Mahony 2008; Tran et al. 2008; Yang and Rao 2008; Chen et al. 2010a, 2010b, 2024; Kuramoto et al. 2011; Mo et al. 2015, 2022; Wang et al. 2015; Pham et al. 2016a, 2016b; Li et al. 2018, 2024, 2025; Shen et al. 2020; Liu et al. 2021; Luo et al. 2021; Zhang et al. 2021; Lin et al. 2022; Song et al. 2023, 2025; Kilunda et al. 2025).

## Results

**Figure 1.**
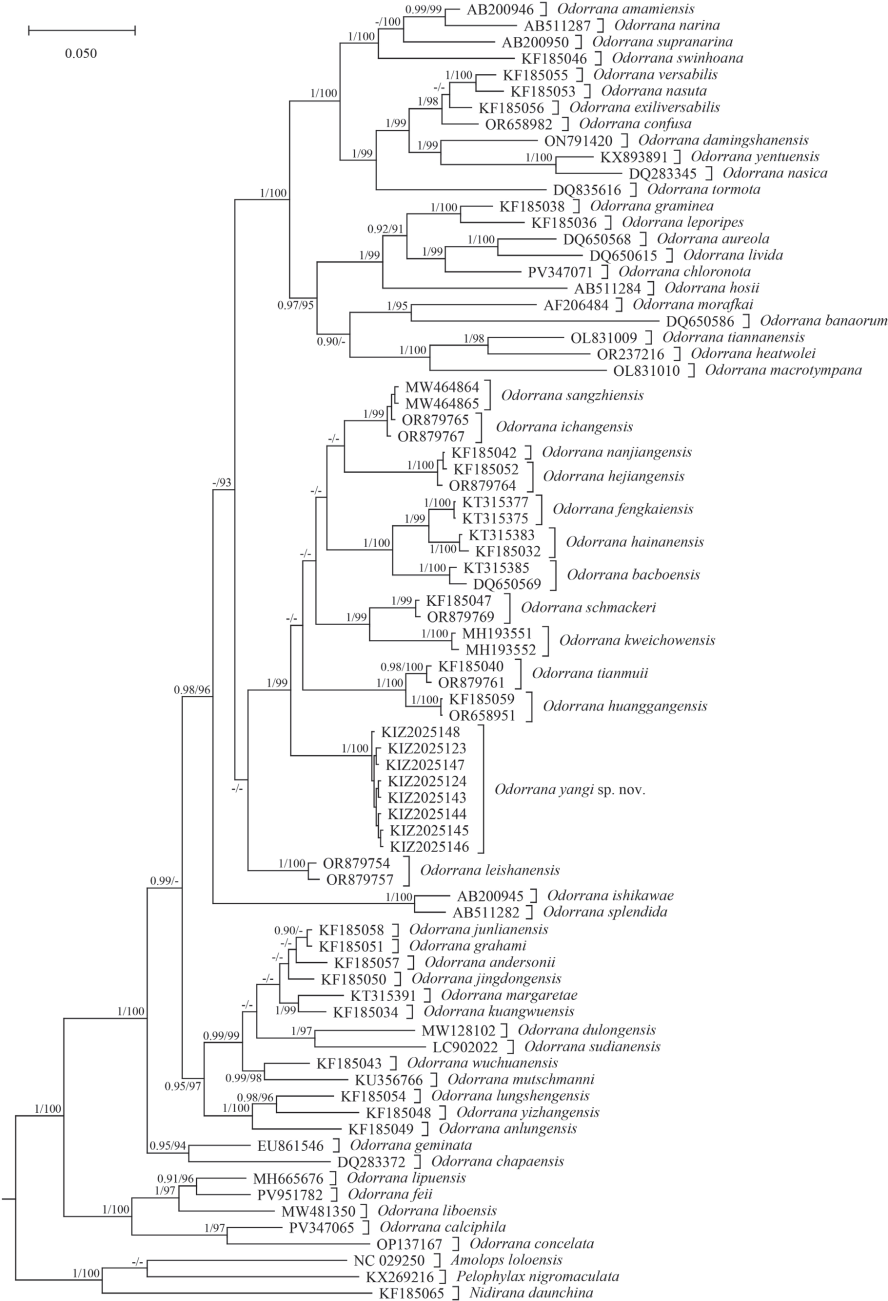
Bayesian-inference tree of the genus *Odorrana* based on 16S sequences. Numbers before slashes indicate Bayesian posterior probabilities (≥0.90 retained) and numbers after slashes indicate the ultrafast bootstrap supports from maximum-likelihood analysis (≥90 retained).

The alignment of the sequences contained 1100 nucleotides, of which 558 sites were conserved and 532 sites were variable, including 376 parsimony-informative sites. Bayesian-inference and maximum-likelihood phylogenetic trees were essentially consistent. The sequences of the specimens from Funing, Yunnan, China, formed a distinct lineage sister to the clade composed of *Odorrana bacboensis* (Bain, Lathrop, Murphy, Orlov & Ho, 2003), *O. fengkaiensis*, *O. hainanensis* Fei, Ye & Li, 2001, *O. hejiangensis* (Deng & Yu, 1992), *O. huanggangensis* Chen, Zhou & Zheng, 2010, *O. ichangensis*, *O. nanjiangensis* Fei, Ye, Xie & Jiang, 2007, *O. sangzhiensis*, *O. schmackeri*, and *O. tianmuii* Chen, Zhou & Zheng, 2010 with strong supports (Fig. 1). The genetic divergences (uncorrected *p*-distance) between the sequences of the specimens from Funing and the sequences of other species of the genus ranged from 3.6% to 14.2% (Suppl. material 1).

### Taxonomy

Our molecular analyses demonstrated that the population from Funing formed a distinct clade in the genus *Odorrana* that exhibits significant genetic divergence of the 16S sequences from other members of the genus. Morphological comparisons further differentiate the Funing population from other species of the genus. Based on these data, we consider that the newly collected specimens from Funing represent an unnamed species of *Odorrana*. Therefore, we formally describe this new species below.

#### Odorrana yangi sp. nov.

Taxon classificationAnimaliaAnuraRanidae

D1CE6E9B-B57C-536C-AF63-9BD2D669A134

https://zoobank.org/D506D272-B112-4417-830E-198C100A350E

Figs 2, 3, 4

##### Type material.

***Holotype***. • KIZ2025143, adult male, collected by Chao Bu on 5 April 2025 from Muba Village, Tianpeng Town, Funing County, Wenshan Prefecture, Yunnan Province, China (23°22'8"N, 105°34'3"E, 710 m a.s.l.). ***Paratypes***. • KIZ2025123, adult female, • KIZ2025124 and KIZ2025144–KIZ2025148, six adult males, all the same collection data as that of the holotype.

**Figure 2.**
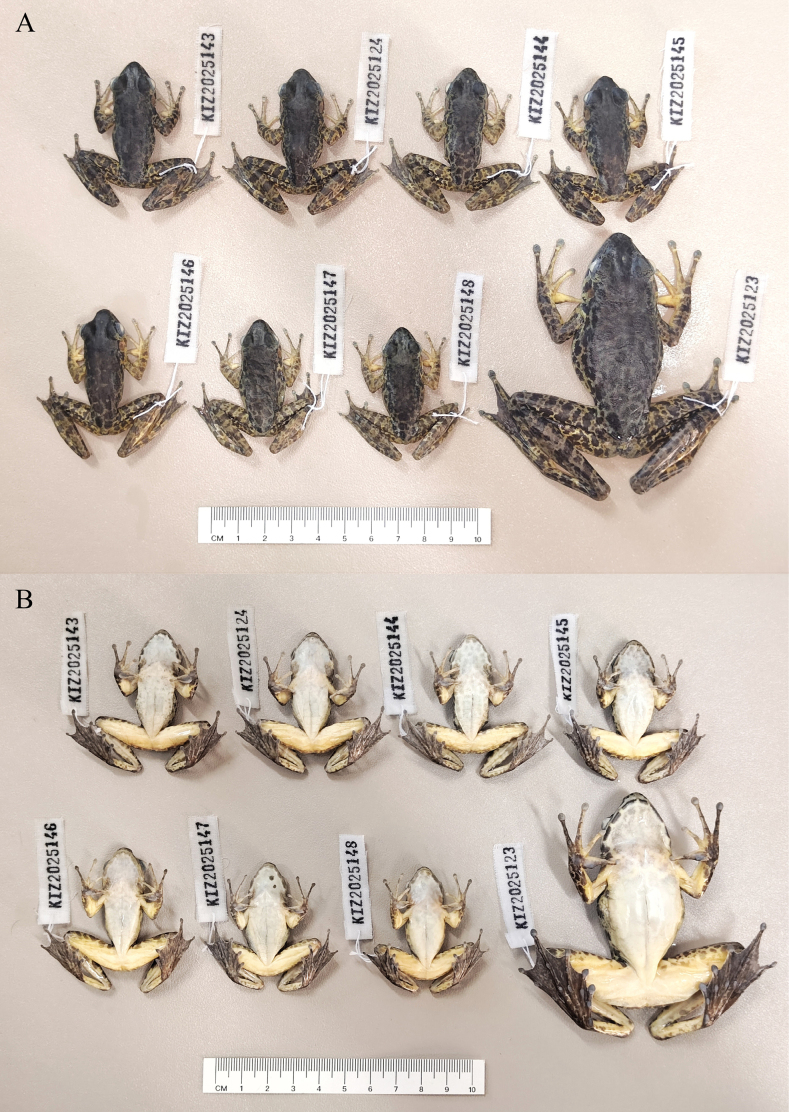
The type specimens of *Odorrana yangi* sp. nov. in in preservative. **A**. Dorsal view; **B**. Ventral view.

**Figure 3.**
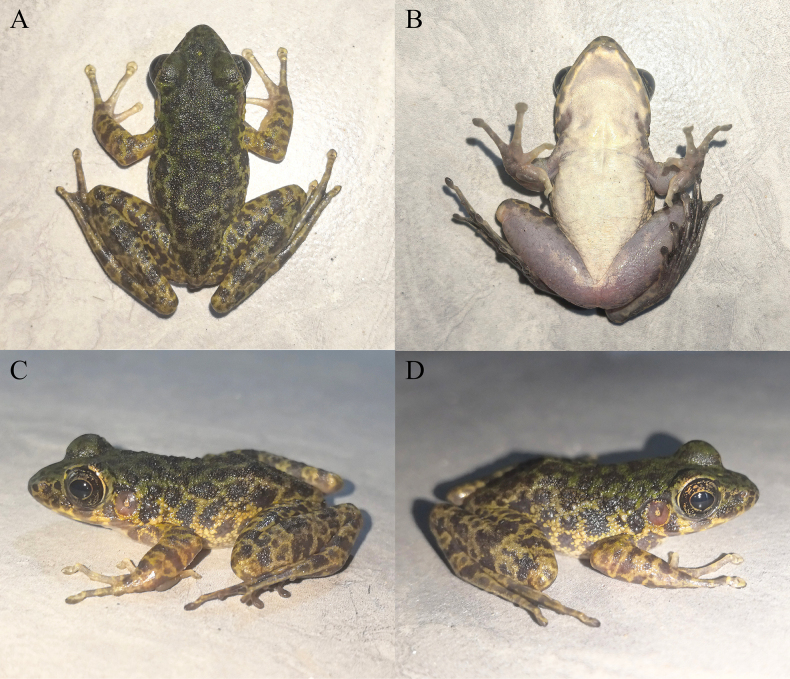
The holotype (KIZ2025143) of *Odorrana yangi* sp. nov. in life. **A**. Dorsal view; **B**. Ventral view; **C**. Left view; **D**. Right view.

**Figure 4.**
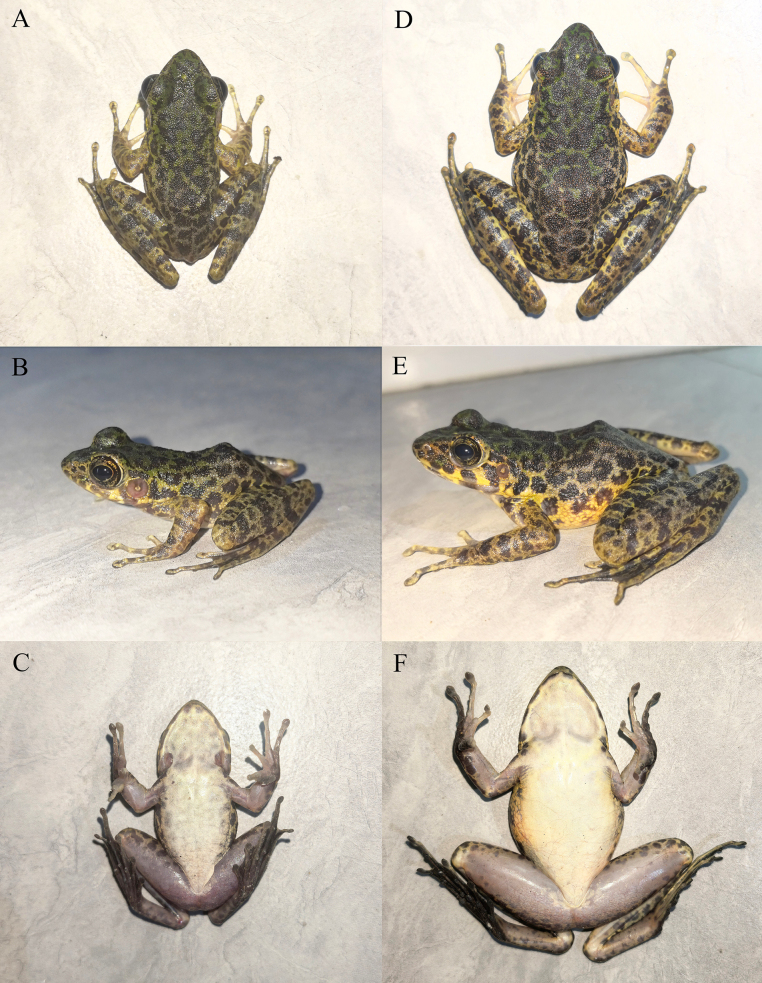
The paratypes of *Odorrana yangi* sp. nov. in life. **A**. Dorsal view of the male (KIZ2025144); **B**. Lateral view of the male (KIZ2025144); **C**. Ventral view of the male (KIZ2025144); **D**. Dorsal view of the female (KIZ2025123); **E**. Lateral view of the female (KIZ2025123); **F**. Ventral view of the female (KIZ2025123).

##### Etymology.

The specific epithet is in commemoration of the renowned Chinese herpetological taxonomist Prof. Dr Datong Yang, who recently passed away. We suggest “Yang’s Odorous Frog” as the common English name and “杨氏臭蛙” as the common Chinese name.

##### Diagnosis.

Body size small in males (SVL 43.5–46.9 mm), females approximately two times size of males; head length greater than head width; nostril closer to tip of snout than to eye; tympanum relatively large in males (TD/ED 0.53–0.63); heels overlap when hindlimbs flexed at right angles to axis of body, tibiotarsal articulation reaching tip of snout when hindlimb stretched forward; relative lengths of fingers III > IV > I > II; dorsal surface relatively smooth, skin on dorsum shagreened, with some indistinct flat tubercles; dorsolateral fold absent; anterior dorsum green with evenly distributed, small, irregular-shaped, black blotches, posterior dorsum greyish brown with evenly distributed, large, irregular-shaped, black blotches; ventral surface white without distinct spots or patches; external vocal sacs and nuptial pads present in adult males.

##### Description of holotype.

Adult male, body size small (SVL 46.4 mm); head length greater than head width (HL/HW 1.12); snout obtuse, pointed in dorsal view and rounded in profile, projecting beyond lower jaw; nostril closer to tip of snout than to eye (NED/NSD 1.15); canthus rostralis distinct, blunt; loreal region concave; internarial distance greater than interorbital distance (IND/IOD 1.49); snout length greater than eye diameter (SL/ED 1.17); pineal body distinct; supratympanic fold distinct; eye moderate (ED/HDL 0.38); tympanum relatively large (TYD/HDL 0.20, TYD/ED 0.53), round; vomerine teeth distinct, two oblique ridges between two choanae; choanae small, rounded; tongue large, cordiform, posterior notched; vocal sac openings small, on floor of mouth in each corner; external vocal sacs below angle of mouth on each side.

Forelimb robust; fingers long, relative length III > IV > I > II; tips of fingers I and II slightly expanded, tips of fingers III and IV obviously expanded into disks; lateral fringes and webbing on fingers absent; subarticular tubercles small and distinct, oval, formula 1, 1, 2, 2; supernumerary tubercles absent; a large oval thenar tubercle on ventral finger I; metacarpal tubercles indistinct; nuptial pad on dorsal finger I.

Hindlimb long (HLL/SVL 1.75), tibia longer than thigh (TL/THL 1.06); toes moderately long, relative length IV > V > III > II > I; all toe tips expanded into disks; fringes on out edges of toes I and VI present; toe webbing formula I1–11/3II1–1½III1–2IV2–1V; subarticular tubercles small and distinct, rounded, formula 1, 1, 2, 3, 2; supernumerary tubercles absent; inner metatarsal tubercle small, oval; outer metatarsal and tarsal fold absent.

Dorsal skin shagreened with some indistinct flat tubercles, skin on flanks slightly granular, dorsolateral fold absent, ventral skin smooth.

##### Colouration of holotype in life.

Dorsal head and anterior dorsum green with evenly distributed, small, irregular-shaped, black blotches, gradually transitioning to yellowish brown with evenly distributed, large irregular-shaped, black blotches posteriorly; dorsal limbs yellowish brown, with brownish-black transverse stripes; upper lib yellow with brownish-black vertical stripes; tympanum flesh-coloured, translucent, with a small, opaque, light-yellow dot at centre; iris light copper-coloured, with black mesh-like pattern; flank yellow, with some large, irregular-shaped, brownish-black spots; ventral surface of head and body white; vocal sac flesh-coloured; ventral surface of limbs light purple.

##### Variations.

Morphological measurements of all type specimens are given in Table 2 and the photographs of two paratypes in life are shown in Fig. 4. The male paratypes are similar to the holotype in morphology and coloration except for the pineal body is almost invisible in some individuals. The female paratype is similar to the males in coloration but significantly larger than the males in body size. In addition, the forelimbs are relatively slender, and the nuptial pad and vocal sac are absent in the female paratype.

**Table 2.** Morphological measurements (mm) of the type specimens of *Odorrana yangi* sp. nov.

	KIZ2025143 Male Holotype	KIZ2025124 Male Paratype	KIZ2025144 Male Paratype	KIZ2025145 Male Paratype	KIZ2025146 Male Paratype	KIZ2025147 Male Paratype	KIZ2025148 Male Paratype	KIZ2025123 Female Paratype
SVL	46.4	46.8	46.5	46.9	46.7	43.5	43.7	80.5
HDL	17.6	18.4	18.0	18.2	17.7	16.4	16.8	29.0
HDW	15.7	16.6	16.4	16.8	15.9	15.2	15.0	27.7
SL	7.7	8.3	8.4	7.7	7.5	7.0	7.3	13.6
NED	3.9	4.4	4.2	4.0	4.2	3.6	4.0	7.6
NSD	3.4	3.8	3.7	3.3	3.4	3.0	3.2	5.9
IND	5.5	5.5	5.6	5.6	5.5	4.9	5.1	8.5
ED	6.6	6.7	6.5	6.5	6.6	5.7	5.8	9.1
IOD	3.7	3.4	3.5	3.6	3.7	3.7	3.6	6.2
UEW	4.8	4.8	4.6	4.6	4.5	4.0	4.3	6.3
TYD	3.5	3.8	3.9	4.0	3.9	3.6	3.3	3.8
LAL	23.1	23.9	24.1	24.4	23.8	23.4	23.2	41.6
ML	15.1	15.4	15.5	15.9	14.7	14.6	14.3	25.0
HLL	81.2	83.1	85.6	86.1	84.2	82.2	79.1	152.0
THL	24.0	24.2	23.3	24.6	24.6	23.5	22.8	43.7
TL	25.5	26.6	26.8	26.8	27.0	25.1	25.5	49.3
TFL	36.4	37.2	38.4	37.9	36.7	36.9	35.1	67.6
FL	25.4	25.9	26.2	26.3	24.9	25.0	23.3	45.9

##### Natural history.

The specimens of the new species were all collected on the rocks or tree branches along a large river at night. Banks of the river are karst landforms. No eggs or tadpoles were found during the survey. *Odorrana tiannanensis* (Yang & Li, 1980) was found to be distributed sympatric with the new species.

##### Distribution.

**Figure 5.**
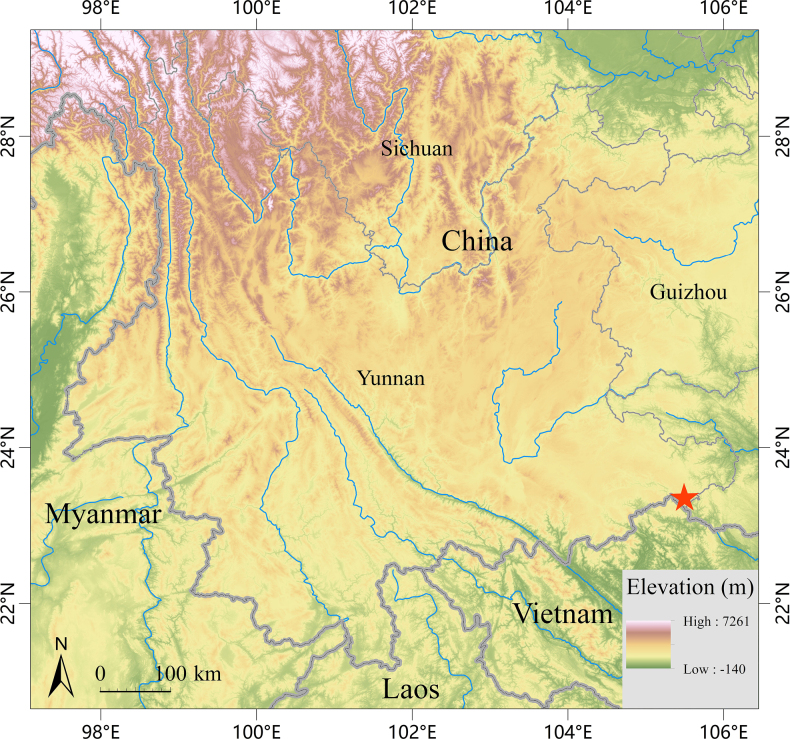
Map showing the type locality (red star) of *Odorrana yangi* sp. nov. in Funing County, Wenshan Prefecture, Yunnan Province, China.

The new species is currently known only from its type locality in Funing County, Wenshan Prefecture, Yunnan Province, China (Fig. 5).

##### Comparisons.

*Odorrana yangi* sp. nov. differs from *O. absita* (Stuart & Chan-ard, 2005), *O. amamiensis* (Matsui, 1994), *O. aureola* Stuart, Chuaynkern, Chan-ard & Inger, 2006, *O. banaorum* (Bain, Lathrop, Murphy, Orlov & Ho, 2003), *O. bolavensis* (Stuart & Bain, 2005), *O. chloronota* (Günther, 1876), *O. confusa*, *O. damingshanensis*, *O. exiliversabilis* Li, Ye & Fei, 2001, *O. gigatympana* (Orlov, Ananjeva & Ho, 2006), *O. graminea* (Boulenger, 1900), *O. hosii* (Boulenger, 1891), *O. heatwolei* (Stuart & Bain, 2005), *O. indeprensa* (Bain & Stuart, 2006), *O. khalam* (Stuart, Orlov & Chan-ard, 2005), *O. leporipes* (Werner, 1930), *O. livida* (Blyth, 1856), *O. macrotympana* (Yang, 2008), *O. monjerai* (Matsui & Jaafar, 2006), *O. montivaga* (Smith, 1921), *O. morafkai* (Bain, Lathrop, Murphy, Orlov & Ho, 2003), *O. narina* (Stejneger, 1901), *O. nasica* (Boulenger, 1903), *O. nasuta* Li, Ye & Fei, 2001, *O. orba* (Stuart & Bain, 2005), *O. supranarina* (Matsui, 1994), *O. tiannanensis*, *O. tormota* (Wu, 1977), *O. utsunomiyaorum* (Matsui, 1994), *O. versabilis*, and *O. yentuensis* Tran, Orlov & Nguyen, 2008 by dorsolateral folds being absent (vs present).

*Odorrana yangi* sp. nov. differs from *O. andersonii*, *O. bacboensis*, *O. cangyuanensis* (Yang, 2008), *O. chapaensis* (Bourret, 1937), *O. geminata* Bain, Stuart, Nguyen, Che & Rao, 2009, *O. grahami* (Boulenger, 1917), *O. ishikawae* (Stejneger, 1901), *O. jingdongensis* Fei, Ye & Li, 2001, *O. junlianensis* Huang, Fei & Ye, 2001, *O. kuangwuensis* (Liu & Hu, 1966), *O. lungshengensis* (Liu & Hu, 1962), *O. margaretae* (Liu, 1950), *O. nanjiangensis*, *O. mawphlangensis* (Pillai & Chanda, 1977), *O. mutschmanni* Pham, Nguyen, Le, Bonkowski & Ziegler, 2016, *O. swinhoana* (Boulenger, 1903), *O. tianmuii*, and *O. wuchuanensis* (Xu, 1983) by having a smaller body size in males (SVL < 47 mm vs SVL > 48 mm).

*Odorrana yangi* sp. nov. differs from *O. calciphila*, *O. concelata*, *O. feii*, *O. leishanensis* Li, Chen, Su, Liu, Tang & Wang, 2024, *O. liboensis*, and *O. lipuensis* by vocal sacs being present in males (vs absent).

*Odorrana yangi* sp. nov. differs from *O. anlungensis* (Liu & Hu, 1973), *O. hejiangensis*, *O. huanggangensis*, *O. ichangensis*, *O. kweichowensis*, and *O. schmackeri* by tibiotarsal articulation reaching snout tip (vs reaching nostril or between eye and nostril); from *O. dulongensis* by head length being greater than head width (vs head length being smaller than head width); from *O. fengkaiensis* and *O. sangzhiensis* by tibiotarsal articulation reaching snout tip (vs beyond snout tip); from *O. hainanensis* by having a pair of external vocal sacs in males (vs a pair of internal vocal sacs); from *O. splendida* Kuramoto, Satou, Oumi, Kurabayashi, & Sumida, 2011 by tibiotarsal articulation reaching snout tip (vs reaching anterior corner of eye); and from *O. sudianensis* and *O. yizhangensis* Fei, Ye & Jiang, 2007 by having different relative length of fingers (III > IV > I > II vs III > I > IV > II in *O. sudianensis* and III > IV > II > I in *O. yizhangensis*).

## Discussion

Amphibian species of the world (Frost 2026) lists 70 species in the genus *Odorrana* at present, including *O. arunachalensis* Saikia, Sinha & Kharkongor, 2017 and *O. sinica* (Ahl, 1927). In our opinion, these two species do not belong to *Odorrana*. Saikia et al. (2017) described *O. arunachalensis* based solely on morphological data without molecular data. Qi et al. (2019) conducted a detailed comparison between the characteristics of *O. arunachalensis* and those of the genera *Odorrana* and *Nanorana* and concluded that this species should belong to the genus *Nanorana*. We examined the original description and photographs of *O. arunachalensis*, and we consider that it belongs to the genus *Nanorana* and is a member of the *N. maculosa* species group due to its colouration on dorsal surface. Therefore, we agree with Qi et al. (2019) and suggest transferring this species to the genus *Nanorana*.

*Rana sinica* Ahl, 1927 was previously listed as a synonym of *R. livida* by Bourret (1942). Later, Bain et al. (2003) resurrected it as a valid species. Currently, Frost (2026) places *R. sinica* within the genus *Odorrana*. We checked the morphological characteristics of *R. sinica* and the photos of the holotype and found that it should belong to the genus *Amolops* and may be a species of the *A. mantzorum* species group due to its colouration, indistinct tympanum, and the absence of the dorsolateral fold. Since the detailed type locality of *R. sinica* is unknown and there is no available molecular data for this species, we cannot determine its specific taxonomic status. We consider that there are two possibilities, one is that *R. sinica* is a species that has not been rediscovered since it was described, and the other is that *R. sinica* is conspecific with a current species of *Amolops*. If the molecular data of the type specimen of *R. sinica* can be obtained in the future, it will help to solve this puzzle.

According to phylogenetic analysis, *Odorrana yangi* sp. nov. belongs to the *O. schmackeri* species group. Although *Odorrana yangi* sp. nov. shares some common characteristics with other species of this group, such as males being significantly smaller than females, no dorsolateral fold, and males having vocal sacs, it is also significantly different from other species of this group in appearance. The dorsal surface of *Odorrana yangi* sp. nov. is green with some evenly distributed, irregular-shaped, black blotches, making it look like a pattern with green reticulation on a black background, which is somewhat similar to the dorsal colouration of species of the *O. lipuensis* group. Although *Odorrana yangi* sp. nov. does not inhabit in limestone caves, its living area is surrounded by karst rocks. More research is needed to reveal whether this coloration is related to limestone habitat.

The collection site of the new species is not within a nature reserve, however, the banks of the river where this species inhabits are limestone cliffs, which are less disturbed by human activities. The population density of this species is relatively large at the type locality, and although we only found one female individual, we encountered many males. In addition, the collection site of this species is very close to the border between China and Vietnam. It is speculated that this species may also be distributed in the neighbouring Ha Giang Province of Vietnam. Therefore, we consider that this species is not endangered at present. In the future, more field investigations should be conducted to clarify the actual distribution range of the species to assess the conservation status of the species.

## Supplementary Material

XML Treatment for Odorrana yangi

## Additional information

### Use of AI

No use of AI was reported.

### Author ORCIDs

Shuo Liu https://orcid.org/0000-0001-7825-3006

Mian Hou https://orcid.org/0000-0002-3322-6570

Dingqi Rao https://orcid.org/0000-0003-2680-1503

Song Li https://orcid.org/0000-0003-0834-5882

### Data availability

All of the data that support the findings of this study are available in the main text or Supplementary Information.

## Supplementary materials

Supplementary material 1 Supplementary information Data type xls Explanation note Uncorrected *p*-distances between species of *Odorrana* based on 16S sequences. https://binary.pensoft.net/file/1569870This dataset is made available under the Open Database License (http://opendatacommons.org/licenses/odbl/1.0/). The Open Database License (ODbL) is a license agreement intended to allow users to freely share, modify, and use this Dataset while maintaining this same freedom for others, provided that the original source and author(s) are credited. Shuo Liu, Chao Bu, Yanfei Feng, Mian Hou, Dingqi Rao, Song Li
